# *bla*_CTX–M–__1_/IncI1-Iγ Plasmids Circulating in *Escherichia coli* From Norwegian Broiler Production Are Related, but Distinguishable

**DOI:** 10.3389/fmicb.2020.00333

**Published:** 2020-03-05

**Authors:** Solveig Sølverød Mo, Amar Anandrao Telke, Kingsley Oteng Osei, Camilla Sekse, Jannice Schau Slettemeås, Anne Margrete Urdahl, Hanna Karin Ilag, Thongpan Leangapichart, Marianne Sunde

**Affiliations:** ^1^Section for Food Safety and Animal Health Research, Department of Animal Health, Welfare and Food Safety, Norwegian Veterinary Institute, Oslo, Norway; ^2^Faculty of Chemistry, Biotechnology and Food Science, Norwegian University of Life Sciences, Ås, Norway; ^3^Section for Microbiology, Department of Analysis and Diagnostics, Norwegian Veterinary Institute, Oslo, Norway

**Keywords:** plasmid, IncI1, cephalosporin resistance, *Escherichia coli*, broiler, *bla*_CTX–M–1_

## Abstract

*Escherichia coli* carrying *bla*_CTX–M–__1_ mediating resistance to extended-spectrum cephalosporins was recently described as a new genotype in Norwegian broiler production. The aim of this study was to characterize these isolates (*n* = 31) in order to determine whether the emergence of the genotype was caused by clonal expansion or horizontal dissemination of *bla*_CTX–M–__1_-carrying plasmids. All included isolates were subjected to whole genome sequencing. Plasmid transferability was determined by conjugation, and plasmid replicons in the transconjugants were described using PCR-based replicon typing. Plasmid sizes were determined using S1 nuclease digestion. Plasmids in a subset of strains were reconstructed and compared to plasmids from broiler production in other European countries. The isolates belonged to nine different sequence types (STs), with the largest group being ST57 (*n* = 12). The vast majority of *bla*_CTX–M–__1_-carrying plasmids were conjugative. All transconjugants were positive for the IncI1-Iγ replicon, and several also harbored the IncFIB replicon. Highly similar plasmids were present in different *E. coli* STs. Additionally, high similarity to previously published plasmids was detected. A reconstructed plasmid from an ST57 isolate harbored both IncI1-Iγ and IncFIB replicons and was considered to be co-integrated. The presence of one large plasmid was confirmed by S1 nuclease digestion. Our results show that dissemination of *bla*_CTX–M–__1_ in Norwegian broiler production is due to both clonal expansion and horizontal transfer of plasmids carrying *bla*_CTX–M–__1_. The *bla*_CTX–M–__1_/IncI1-Iγ plasmids grouped into two main lineages, namely clonal complex (CC)-3 and CC-7. The genetic diversity at both strain and plasmid level indicates multiple introductions to Norway. We also show that the *bla*_CTX–M–__1_ plasmids circulating in Norwegian broiler production are highly similar to plasmids previously described in other countries.

## Introduction

Since the first description of an *Escherichia coli* displaying extended-spectrum cephalosporin (ESC) resistance in broilers in 2000–2001 (Briñas et al., 2003), numerous reports have underlined the global occurrence of these bacteria in the broiler production (Carattoli, 2008; Smet et al., 2008; Dierikx et al., 2010; Doi et al., 2010; Ewers et al., 2012; Hiroi et al., 2012b). Import of breeding animals is believed to be a contributing factor to the wide dissemination. The first detection of *E. coli* mediating ESC resistance in Norwegian livestock dates back to year 2006. The isolate originated from healthy broilers and harbored the *bla*_TEM–__20_ gene on an IncI1 plasmid (NORM/NORM-VET, 2007; Sunde et al., 2009). In 2011, a selective method for detection of ESC-resistant *E. coli* was implemented in the Norwegian monitoring programme for antimicrobial resistance in bacteria from food, feed and animals (NORM-VET) (NORM/NORM-VET, 2012). Since then, all ESC-resistant *E. coli* isolated from broiler production in the NORM-VET program have displayed an AmpC phenotype due to presence of the *bla*_CMY–__2_ gene (NORM/NORM-VET, 2012, 2013, 2015, 2017). However, an extensive sampling of broiler- and parent flocks during 2016 revealed an additional presence of *E. coli* producing extended-spectrum beta-lactamases (ESBLs), with *bla*_CTX–M–__1_ being the most frequently detected ESBL-encoding gene (Mo et al., 2019 and unpublished data). In other European countries, the *bla*_CTX–M–__1_ gene has been frequently associated with ESBL-producing *E. coli* from the broiler production chain. Several studies have shown that the *bla*_CTX–M–__1_ gene commonly occurs on conjugative IncI1 plasmids (Zurfluh et al., 2014; Smith et al., 2015; Irrgang et al., 2018; Touzain et al., 2018).

The aim of this study was to characterize the *bla*_CTX–M–__1_ harboring *E. coli* isolates in order to determine whether the emergence of this genotype was due to clonal expansion of resistant strains or horizontal transmission of *bla*_CTX–M–__1_-carrying plasmid(s). The *bla*_CTX–M–__1_-harboring plasmids identified were further investigated, and a selection of plasmids were reconstructed and compared to plasmids originating from broiler production in other European countries and from other sources in order to identify successful plasmids.

## Materials and Methods

### Bacterial Isolates

The isolates included were retrieved from a previous study. In short; during the period from May to October 2016 all broiler flocks reared in Norway were investigated for ESC-resistant *Enterobacteriaceae* (*n* = 2110). Approximately 10% of the flocks were positive, of which the majority contained the *bla*_CMY–__2_ gene responsible for ESC resistance. A minor proportion (*n* = 24, 1.1% of total flocks) were positive for *bla*_CTX–M–__1_, and these isolates were further characterized in the present study. Detailed description of the sampling and detection methods is described previously (Mo et al., 2019). The isolates originated from 24 unique broiler flocks reared at 15 different farms. Nine farms had one positive flock, and four farms had two positive flocks, while two farms had three and four positive flocks, respectively. In addition, seven *E. coli* with *bla*_CTX–M–__1_ isolated from parent flocks were included (unpublished data). These isolates originated from three different parent flocks sampled at multiple times during a 4 week period in November–December 2016. One parent flock was positive at one sampling, while the two remaining flocks were positive at three samplings each.

### Susceptibility Testing

The minimum inhibitory concentration (MIC) values of the isolates were determined by broth microdilution (EUVSEC, Sensititre^®^, TREK Diagnostic Systems Ltd., Thermo Fisher Scientific). For quality control, *E. coli* ATCC25922 was tested on a regular basis. Epidemiological cut-off values from EUCAST (European Committee on Antimicrobial Susceptibility Testing) were used to categorize the isolates as susceptible or resistant. MIC values for all broiler isolates (*n* = 24) were available from a previous study (Mo et al., 2019), while isolates originating from parent flocks (*n* = 7) were tested as a part of this study.

### Determination of Phylogroups

Classification of isolates into phylogroups A, B1, B2, or D was performed on all isolates using a previously described multiplex PCR (Doumith et al., 2012). Positive and negative controls were included in each PCR run.

### Conjugation Experiments and Characterization of Transconjugants

To determine the transferability of the *bla*_CTX–M–__1_-carrying plasmids, all isolates were subjected to conjugation experiments in broth as previously described (Mo et al., 2016). *E. coli* DH5α CCUG32825 was used as recipient. Transconjugants were confirmed on the basis of their characteristic colony morphology on blood agar and on lactose-saccharose-bromthymol blue agar (Mo et al., 2016), and PCR was carried out to confirm the presence of *bla*_CTX–M_ (Hasman et al., 2005). Further confirmation of transconjugants included PCR-based replicon typing (PBRT) to determine the replicon type(s) of the transferred plasmids in transconjugants. This was done using the PBRT kit (Diatheva, Fano, Italy) as described by the manufacturer. Positive and negative controls were included in each PCR run. To determine the approximate size of selected *bla*_CTX–M–__1_-carrying plasmids, transconjugants of ST57 isolates (*n* = 12) were subjected to S1 nuclease digestion (Invitrogen^TM^, Carlsbad, CA, United States) of DNA embedded in agarose followed by pulsed-field gel electrophoresis (PFGE) as previously described (Barton et al., 1995).

### DNA Isolation and Sequencing

For Illumina sequencing, DNA was extracted using the QIAamp DSP DNA mini kit (Qiagen, Hilden, Germany) with the QiaSymphony automated extractor system (Qiagen). The DNA yield and purity was measured on a NanoDrop 2000 spectrophotometer (Thermo Fisher Scientific, Waltham, MA, United States) and on a Qubit fluorometer (Invitrogen) using Qubit dsDNA BR Assay kit (Invitrogen). Whole genome sequencing (WGS) was performed on all isolates using the Nextera XT library preparation kit (Illumina, San Diego, CA, United States) and a NextSeq 500 Illumina platform resulting in 150 bp paired-end reads.

To facilitate reconstruction and in-depth characterization of *bla*_CTX–M–__1_-carrying plasmids, nine isolates representing each multilocus sequence type (MLST)/core genome (cg)MLST cluster identified were selected for Pacific Biosciences (PacBio, Menlo Park, CA, United States) long-read sequencing (Table 1). Total genomic DNA was extracted using a Qiagen Genomic-tip 100/G kit (Qiagen). The quality and quantity of DNA was assessed as described in the previous paragraph. The DNA library was prepared using a protocol for single molecule real-time (SMRT) bell^TM^ libraries (PacBio). The library was sequenced on a PacBio Sequel platform using sequel polymerase v3.0, a 10 kb insert library, SMRT cells v3 LR and Sequencing chemistry v3.0 (PacBio).

**TABLE 1:** Overview of characteristics associated with 31 *Escherichia coli* isolates with *bla*_CTX–M–__1_ originating from broiler- (*n* = 24) and parent flocks (*n* = 7, isolates from three flocks) in Norway in 2016.

**Isolate**	**Origin**	**Parent flock**	**Broiler farm**	**Phylo-group**	**MLST**	**Pathogroup (diarrhoeagenic)**	**Plasmid replicon type(s)**	**IncI1 pMLST (clonal complex)**	**Other AMR genes**	**Transferable plasmid**	**Plasmid replicon type(s) in transconjugant**	**PacBio**
2016-40-14272	Broiler		B	D	57		I1, FIB	3 (CC-3)	*sul2*	+	I1,FIB	
2016-40-16262	Broiler		B	D	57		I1, FIB, B/O/K/Z, I2	42 (CC-3)	*sul2*	+	I1,FIB	
2016-40-17200	Broiler		B	D	57		I1, FIB, B/O/K/Z	42 (CC-3)	*Sul2*	+	I1,FIB	
2016-40-19738	Broiler		B	D	57		I1, FIB, B/O/K/Z	42 (CC-3)	*sul2*	+	I1,FIB	
2016-40-14497	Broiler		C	D	57		I1, FIB	3 (CC-3)	*sul2*	+	I1,FIB	
2016-40-17437	Broiler		C	D	57		I1, FIB	3 (CC-3)	*sul2*	+	I1,FIB	+
2016-40-19583	Broiler		C	D	57		I1, FIB	3 (CC-3)	*sul2*	+	I1,FIB	
2016-40-16990	Broiler		D	D	57		I1, FIB	3 (CC-3)	*sul2*	+	I1,FIB	
2016-40-17074	Broiler		D	D	57		I1, FIB	3 (CC-3)	*sul2*	+	I1,FIB	
2016-40-16344	Broiler		G	D	57		I1, FIB	3 (CC-3)	*sul2*	+	I1,FIB	
2016-40-17091	Broiler		H	D	57		I1, FIB	3 (CC-3)	*sul2*	+	I1,FIB	
2016-40-20481	Broiler		M	D	57		I1, FIB, B/O/K/Z	42 (CC-3)	*sul2*	+	I1	
2016-40-19016	Broiler		E	B1	297		I1, FIB, FIC/FII, FIA, B/O/K/Z	IC	*sul2*	+	I1	
2016-40-19138	Broiler		F	B1	297		I1, FIB, FIC/FII, FIA, B/O/K/Z	3 (CC-3)	*sul2*	+	I1	+
2016-40-17381	Broiler		I	B1	297		I1, FIB, FIC/FII, FIA, B/O/K/Z, X1	IC	*sul2*	+	I1,B/O/K/Z	
2016-40-19148	Broiler		J	B1	297		I1, FIB, FIC/FII, FIA, B/O/K/Z	IC	*sul2*	+	I1,B/O/K/Z	
2016-40-19970	Broiler		K	B1	297		I1, FIB, FIC/FII, FIA, B/O/K/Z	IC	*sul2*	+	I1,B/O/K/Z	
2016-40-21210	Broiler		N	B1	297		I1, FIB, FIC/FII, FIA, B/O/K/Z	3 (CC-3)	*sul2*	+	I1	
2016-40-21270	Broiler		O	B1	297		I1, FIB, FIC/FII, FIA, B/O/K/Z	IC	*sul2*	+	I1	
2016-40-14263	Broiler		A	A	752	EPEC	I1, FIB, FIC/FII	7 (CC-7)	*aph*	-	NA	+
2016-40-20703	Broiler		A	A	752	EPEC	I1, FIB, FII	7 (CC-7)	*aph*	-	NA	
2016-40-23575	Parent	3		A	752	EPEC	I1, FIB, FII, FIC, I2	7 (CC-7)	*aph*	-	NA	
2016-40-22440	Parent	1		A	48		I1, FIB, FII, HI1B	3 (CC-3)	*sul2*, *tetA*	+	I1	+
2016-40-23572	Parent	1		A	48		I1, FIB, FII, HI1B	3 (CC-3)	*sul2*, *tetA*	+	I1	
2016-40-24053	Parent	1		A	48		I1, FIB, FII, HI1B, X4	3 (CC-3)	*sul2*, *tetA*	+	I1	
2016-40-22638	Parent	2		A	1638		I1, FIB, FII	3 (CC-3)	*aadA*, *dfrA*, *sul1*, *sul2*, *tetA*	+	I1	+
2016-40-23574	Parent	2		A	1638		I1, FIB, FII	3 (CC-3)	*aadA*, *dfrA*, *sul1*, *sul2*, *tetA*	+	I1	
2016-40-21249	Broiler		F	B1	162		I1, FIB, FII, X1	3 (CC-3)	*sul2*	+	I1	+
2016-40-21254	Broiler		E	A	10		I1	3 (CC-3)	*sul2*	+	I1	+
2016-40-24003	Parent	2		A	1251		I1, FIB, FII, HI1B	3 (CC-3)	*aadA*, *dfrA*, *sul1*, *sul2*, *tetA*	+	I1	+
2016-40-20426	Broiler		L	D	641		I1, FIB, FII, FIA	3 (CC-3)	*sul2*	+	I1	+

*EPEC, enteropathogenic *Escherichia coli*; IC, incomplete sequence type; AMR, antimicrobial resistance; NA, not applicable.*

The sequencing was performed by the Norwegian Sequencing Centre.

### Sequence Analysis and Characterization

The Bifrost pipeline (Lagesen, 2020)^1^ was used for initial analysis of fastq data from the Illumina sequencing. Quality control of the raw reads was done using FastQC^2^, and multiQC (Ewels et al., 2016) was used to merge the results. Furthermore, Trimmomatic (Bolger et al., 2014) was used to trim the reads, and bbduk^3^ removed PhiX. Thereafter, *de novo* assembly was done using SPAdes (Bankevich et al., 2012). Only contigs longer than 500 bp were withheld in the assemblies. Assemblies were polished and improved using Pilon (Walker et al., 2014), and QUAST (Gurevich et al., 2013) was used to evaluate the assemblies using *E. coli* K-12 substr. MG1655 (accession number NZ_CP027060.1) as reference. The sequence types (STs) were predicted by the ARIBA (antimicrobial resistance identification by assembly) tool (Hunt et al., 2017) using the Achtman scheme (Wirth et al., 2006). ARIBA was also used to predict the acquired antimicrobial resistance (AMR) genes present using the ResFinder database (Zankari et al., 2012), and virulence genes using the VirulenceFinder database (Joensen et al., 2014). Furthermore, we used chewbacca (Silva et al., 2018) to perform cgMLST using the scheme available from Enterobase^4^. Vizualisation of cgMLST data was done in R version 3.5.2 (R Core Team, 2019). Plasmid replicons and pMLST profiles were determined using PlasmidFinder (Carattoli et al., 2014) and pMLST 2.0 (Carattoli et al., 2014).

Long reads were demultiplexed using the barcoding pipeline on SMRT Link (v6.0.0.47841, SMRT Link Analysis Services and GUI v6.0.0.47836) with 40 as minimum barcode score. Read mapping was carried out to assess the quality of data and coverage using Minimap2 (Li, 2018). Illumina reads were used as reference in the filtlong tool^5^ to produce a better subset of PacBio reads. Hybrid assemblies were generated using Unicycler version v0.4.7 (Wick et al., 2017). Bandage (Wick et al., 2015) was used to assess the quality of assemblies. Circularized plasmid contigs were investigated using BLASTn^6^, 15th August 2019, and the curated complete plasmid sequences were annotated using Prokka version 1.13.7 (Seemann, 2014). Annotations were analyzed and curated using Artemis (Carver et al., 2005) and compared using Easyfig (Sullivan et al., 2011). Sequence differences in the genes encoding replication (*inc*), partition (*parAB*), and entry exclusion (*traY-excA*) can be used to distinguish between plasmids of the IncI1 and IncIγ groups (Takahashi et al., 2011). Thus, the nucleotide sequence of the *inc* gene, and amino acid sequence of parAB and ExcA were compared to the corresponding regions in R64 (accession number AP005147) and R621a (accession number AP011954.1) to further analyze the plasmid replicon types.

Reconstructed plasmids were compared to IncI plasmids from broilers in the Netherlands, Switzerland, France, and Denmark available from GenBank.

Complete plasmid sequences are available in GenBank; MN419430 (p17437), MN419431 (p19138), MN419432 (p21254), MN419433 (p20426), MN419434 (21249), MN419435 (p14263), MN419436 (p22440), MN419437 (p22638), and MN419438 (p24003). Raw reads for the 31 *E. coli* isolates are available from ENA (bioproject number PRJEB45077).

## Results

### Strain Characterization

**FIGURE 1:**
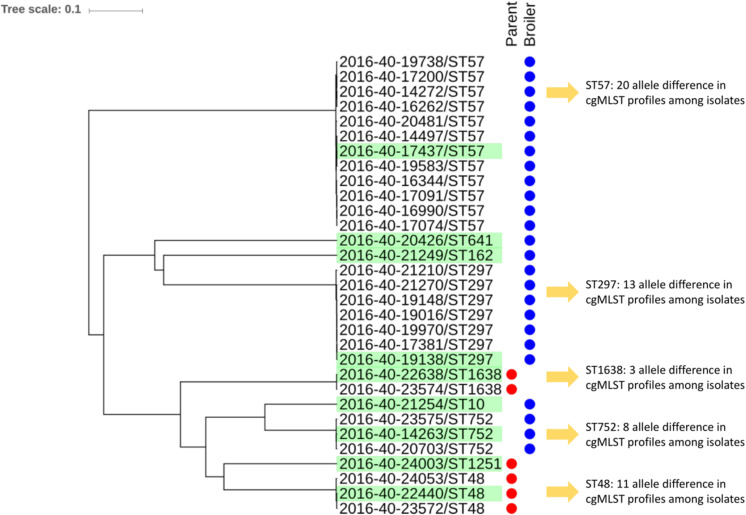
Clustering of 31 *Escherichia coli* isolates with *bla*_CTX–M–__1_ isolated from Norwegian broiler production in 2016 based on core genome MLST using the scheme available from Enterobase. Isolates selected for long read sequencing are highlighted in green.

An overview of the strain characteristics is presented in Table 1. Most isolates belonged to phylogroup D (*n* = 13), followed by A (*n* = 10), and B1 (*n* = 8). None of the isolates were characterized as B2 (Table 1). The 31 *E. coli* isolates belonged to nine different STs, with ST57 (*n* = 12) and ST297 (*n* = 7) being the most common. Each MLST group represented one cluster in the cgMLST analysis (Figure 1). On broiler farm A, *E. coli* ST752 was detected twice, differing by eight alleles. On broiler farm B, ST57 isolates were detected four times, two isolates with identical cgMLST profiles, and only three allele differences between all four isolates. Two ST57 isolates with identical cgMLST profiles and a third isolate differing by only one allele were detected from farm C. All ST297 isolates originated from different farms. Among these, two shared identical cgMLST patterns, and the maximum allele difference was 13.

Table 2 presents the results from the antimicrobial susceptibility testing. Six isolates displayed multidrug resistance (MDR, resistance to three or more antimicrobial classes, Table 1). All isolates were cephalosporin-resistant, and most were co-resistant to at least one antimicrobial class. The most commonly detected co-resistance was to sulfonamides (*n* = 27, 87%), followed by tetracycline (*n* = 6, 19%), and trimethoprim (*n* = 3, 10%). Resistance to the critically important antimicrobials carbapenem and colistin was not observed. WGS data confirmed the presence of *bla*_CTX–M–__1_ in all isolates. Additional AMR genes detected in the isolates are shown in Table 1. All three ST752 isolates harbored the *eae* gene, and were categorized as enteropathogenic *E. coli* (EPEC) (Table 1; Kaper et al., 2004). The remaining isolates did not harbor virulence genes associated with an *E. coli* pathogroup. An overview of all virulence genes detected is available in Supplementary Table 1.

**TABLE 2:** Minimum inhibitory concentrations (MICs) and antimicrobial resistance in *Escherichia coli* harboring *bla*_CTX–M–__1_ (*n* = 31) isolated from broiler- and parent flocks in Norway during 2016.

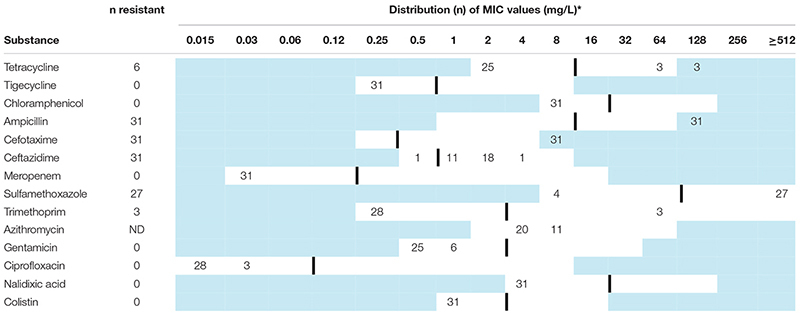

*Bold vertical lines denote epidemiological cut-off values for resistance. ND, cut-off not defined by EUCAST. White fields denote range of dilutions tested for each antimicrobial agent. MIC values higher than the highest concentration tested are given as the lowest MIC value above the range. MIC values equal to or lower than the lowest concentration tested are given as the lowest concentration tested.

### Plasmid Characterization

**FIGURE 2:**
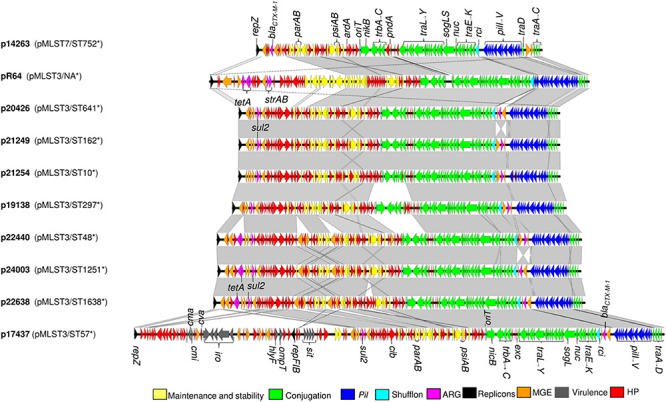
Genetic organization of plasmids p14263, p20426, p21249, p21254, p19138, p22440, p24003, p22638, and p17437 including comparison to the IncI1 reference plasmid R64 (accession number AP005147). Areas shaded gray indicate homologus regions, nucleotide identity threshold >95%. ARG, antimicrobial resistance genes; MGE, mobile genetic elements; HP, hypothetical proteins; NA, not available; *, sequence type of host strain.

The *bla*_CTX–M–__1_ gene was located on conjugative plasmids in 28 of the 31 isolates (90%) (Table 1). This was confirmed by the presence of a single replicon type in most transconjugants (Table 1), and the presence of a complete transfer region on all fully sequenced pMLST3 plasmids (Figure 2; Carattoli et al., 2018). The *bla*_CTX–M–__1_-carrying plasmids in the three remaining isolates were not able to conjugate into *E. coli* DH5α.

PlasmidFinder confirmed the presence of IncI1-Iγ replicons in all isolates. Additional replicons like FIB and B/O/K/Z were also present in subsets of the isolates (Table 1). pMLST revealed that 19 of the IncI1-Iγ plasmids were ST3, three were ST7 and four were ST42. ST3 and ST42 group into the same clonal complex (CC), namely CC-3. Five IncI1-Iγ plasmids had an incomplete ST (IC) (Table 1). PBRT showed that all transconjugants harbored IncI1 replicons, and WGS data confirmed that *bla*_CTX–M–__1_ was located on IncI1-Iγ plasmids in all isolates. Some transconjugants also harbored FIB and B/O/K/Z replicons as shown in Table 1.

**FIGURE 3:**
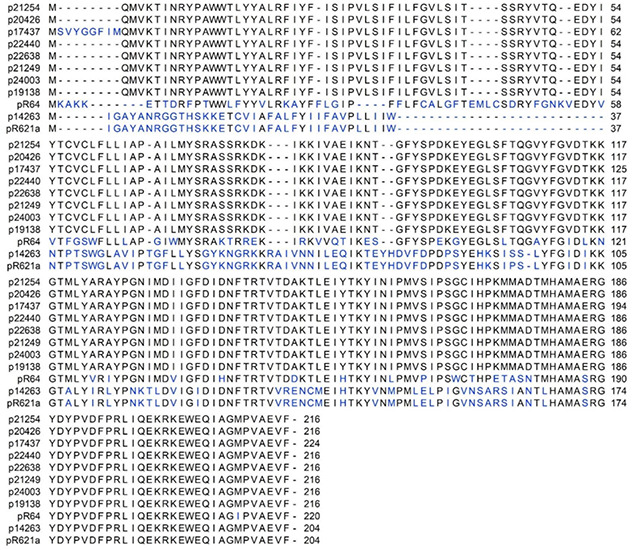
Alignment of the amino acid sequences of ExcA of the nine IncI1-Iγ plasmids reconstructed in this study, IncI1 prototype R64 (accession number AP005147) and IncIγ prototype R621a (accession number AP011954.1). Gaps are marked by dashes, and amino acid differences are shown in blue.

The genetic organization of the nine reconstructed IncI1-Iγ plasmids is visualized in Figure 2. The backbone structures of the plasmids were similar to R64. The *traABCD* regulatory gene cluster was located at the distal end of the type IV pili locus region in ST3 plasmids (Figure 2). In the re-constructed ST7 plasmid, the *traC* gene was disrupted by insertion of the ISS*oEn3* transposase. The shufflon consisted of a number of invertible DNA segments (Table 3), and was located at the proximal end of the type IV pili locus region. Genes encoding addiction systems, namely *relBE*, *phd/doc*, and *pndAC* were present on all plasmids (Table 3). Some differences were observed in the regions encoding *trbABC* and *nikB* in p19138 compared to the other ST3 plasmids (Figure 2). In most plasmids, the nucleotide sequence of the *inc* gene was identical to that found in the R64 plasmid, while three deletions in the 3’ end was present in one plasmid (p17437) (Supplementary Figure 1). The ParAB amino acid sequences were also identical to that of R64 in all plasmids (Supplementary Figures 2, 3). In seven of our plasmids, the amino acid sequence of ExcA had 64% sequence identity compared to R64, and 38% sequence identity compared to R621a. p17437 differed by eight amino acids at the N-terminal compared to the rest of our plasmids. The amino acid sequence of ExcA in the ST7 plasmid (p14263) had 99.5% sequence identity to the one in R621a (Figure 3). The results show that our plasmids cannot be unambiguously grouped as belonging to the I1 or Iγ, as they show intermediate evolutionary levels between these replicon types. Thus, we refer to them as IncI1-Iγ.

**TABLE 3:** Overview of characteristics associated with IncI1-Iγ plasmids carrying *bla*_CTX–M–__1_ in nine *Escherichia coli* isolated from Norwegian broilers and parent flocks in 2016.

**Isolate**	**MLST**	**Plasmid**	**pMLST**	**Estimated plasmid size (bp)**	**Other AMR genes**	**Shufflon regions**	**Virulence determinants**
2016-40-14263	ST752	p14263	ST7	91030	ND	CC’B’BA’A	ND
2016-40-20426	ST641	p20426	ST3	102183	*sul2*	B’BC’CA’A	*cib*
2016-40-21249	ST162	p21249	ST3	102184	*sul2*	BB’C’CA’A	*cib*
2016-40-21254	ST10	p21254	ST3	102373	*sul2*	BB’CC’A’A	*cib*
2016-40-19138	ST297	p19138	ST3	106298	*sul2*	BB’CC’A’A	*cib*
2016-40-22440	ST48	p22440	ST3	115586	*sul2, tetA*	B’BCC’A’A	*cib*
2016-40-24003	ST1251	p24003	ST3	116056	*sul2, tetA*	CC’BB’A’A	*cib*
2016-40-22638	ST1638	p22638	ST3	118243	*sul2, tetA*	B’BCC’A’A	*cib*
2016-40-17437	ST57	p17437	ST3	168655	*sul2*	B’BC’CA’A	*cib, cma, cva, sit, iro, hlyF, ompT*

*AMR, antimicrobial resistance; ND, not detected.*

Highly similar plasmids belonging to ST3 were detected in *E. coli* ST1638, ST1251 and ST48. These plasmids ranged in size from 115586-118243 bp and carried the *sul*2 and *tet*A genes inserted as a part of the accessory module. In addition, somewhat smaller IncI1-Iγ/ST3 plasmids, ranging from 102183-102373 bp and carrying only *sul2* in addition to *bla*_CTX–M–__1_, were present in ST641, ST162, and ST10 (Table 2). On all IncI1-Iγ/ST3 plasmids, *bla*_CTX–M–__1_ was linked with IS*Ecp1*, and located in close proximity to the shufflon. In contrast, the IS*Ecp1*-*bla*_*CTX*__–M–__1_ region was inserted in the accessory module in the IncI1-Iγ/ST7 plasmid. All IncI1-Iγ/ST3 plasmids harbored the *cib* gene encoding a colicin (Table 2).

**FIGURE 4:**
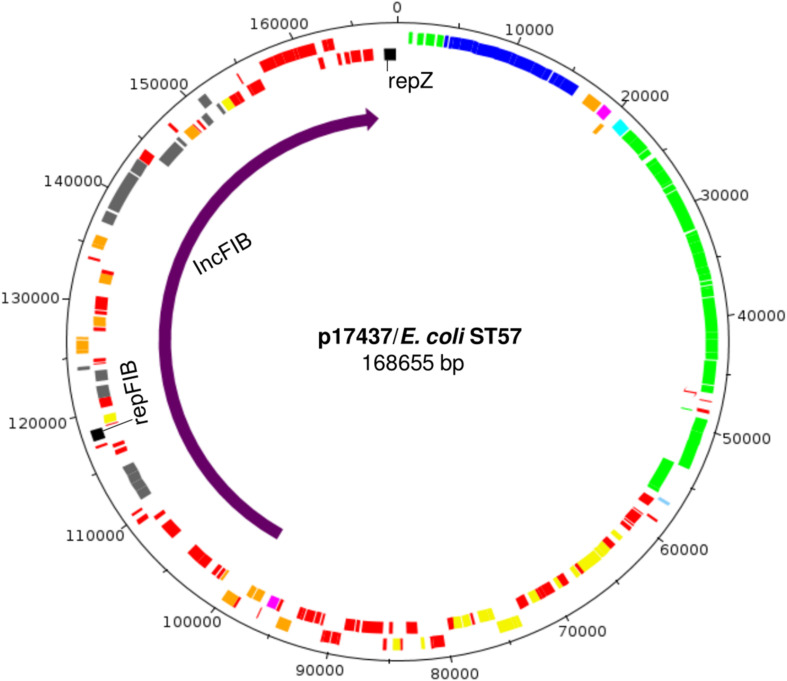
Genomic map of the co-integrated plasmid p17437 harboring both IncI1-Iγ and IncFIB replicons. The region indicated by the purple arrow is the IncFIB specific region, while the remaining part is the IncI1-Iγ specific region. Positions of genes are indicated by color, and their meaning is as follows; yellow, maintenance and stability cluster; green, transfer locus; blue, pilus; black, plasmid replicons; aqua, shufflon; pink, antimicrobial resistance genes; orange, transposome; gray, virulence genes; red, hypothetical proteins.

In one reconstructed ST3 plasmid, two replicons were detected, namely IncI1-Iγ and IncFIB (p17437, Table 3). Further characterization implied that this IncI1-Iγ/IncFIB co-integrated plasmid consisted of a complete IncI1-Iγ plasmid and a fraction of an IncFIB plasmid (Figures 2, 4). Several virulence determinants, including *sit*, *iroN* and *hlyF*, were encoded on the IncFIB fraction of the plasmid. The total length of the co-integrated plasmid was 168655 bp (Figures 2, 4 and Table 3). S1 nuclease digestion confirmed the presence of the plasmid in all ST57 transconjugants that were positive for both the IncI1-Iγ and IncFIB replicons. One ST57 transconjugant had only the IncI1-Iγ replicon, and harbored a smaller plasmid (Supplementary Figure 4).

Comparison with IncI1-Iγ plasmids originating from broiler production in other European countries revealed close similarity to plasmids characterized in this study (Supplementary Figures 5–7). Our data show clonal dissemination of *E. coli* STs carrying *bla*_CTX–M–__1_, but also horizontal transfer of highly similar *bla*_CTX–M–__1_ carrying plasmids in Norwegian broiler production is illustrated in Supplementary Figure 8.

## Discussion

The isolates investigated represent a unique collection including all *bla*_CTX–M–__1_ isolates originating from Norwegian broilers sampled during a six month period (May–October) and Norwegian parent flocks sampled during a four week period (medio November–medio December) in 2016. Thus, it gives us substantial knowledge regarding different *E. coli* STs and plasmids circulating, and enables evaluation of plasmid- and ST flux and epidemiology over a prolonged time period in the broiler/parent population at national level.

Nine different *E. coli* STs were detected among the 31 isolates included, underlining some genetic diversity among the isolates. However, ST57 and ST297 were detected from several flocks and farms indicating possible clonal dissemination of certain *E. coli* STs with *bla*_CTX–M–__1_ in Norwegian broiler production. This is supported by identical or highly similar cgMLST profiles among the isolates. All three isolates from parent flock 1 belonged to ST48 and clustered together in the cgMLST analysis, differing by 11 alleles. On the contrary, one of the three isolates from parent flock 2 belonged to ST1251, while the other two belonged to ST1638. However, the *bla*_CTX–M–__1_ carrying plasmids in these three isolates were highly similar, possibly indicating horizontal plasmid transfer between different *E. coli* STs. On six of the 15 broiler farms two or more flocks were positive for *bla*_CTX–M–__1_. This could be due to on-farm persistence of ESC-resistant bacteria as previously suggested (Hiroi et al., 2012a; Dierikx C. et al., 2013; Laube et al., 2013; Agersø et al., 2014; Nilsson et al., 2014).

ST752 isolates (*n* = 3) included one isolate from a parent flock and two isolates from broiler flocks. These isolates differed by only eight alleles in their cgMLST profiles. This could indicate clonal dissemination of ESBL-producing *E. coli* in the production pyramid, which has also been proposed by others (Sunde et al., 2009; Dierikx C.M. et al., 2013; Nilsson et al., 2014; Zurfluh et al., 2014). Unfortunately, we did not have information regarding which parent flocks supplied the broiler flocks included in this study. Thus, we cannot confirm the presence of related isolates in broiler flocks and their supplying parent flock(s) based on our data.

*E. coli* carrying *bla*_CTX–M–__1_ on transferable IncI1 plasmids is also commonly occurring in European broiler production (Börjesson et al., 2013a, b; Zurfluh et al., 2014; DANMAP, 2015; Smith et al., 2015; Irrgang et al., 2018; Touzain et al., 2018). Most of the IncI1-Iγ plasmids in our study were identified as ST3 (*n* = 19), which is also a common ST in broiler production in other countries (Zurfluh et al., 2014; Smith et al., 2015; Baron et al., 2018; Irrgang et al., 2018), and the most commonly reported IncI1-Iγ plasmid associated with *bla*_CTX–M–__1_ in Europe (Carattoli et al., 2018). We detected highly similar IncI1-Iγ/ST3 plasmids in different *E. coli* STs, indicating that the plasmids are able to disseminate horizontally in the *E. coli* population. Further comparison of a selection of IncI1-Iγ/ST3 plasmids from broiler production in Norway and other European countries, including the Netherlands, Switzerland, France, and Denmark (Brouwer et al., 2014; Wang et al., 2014; Zurfluh et al., 2014; Touzain et al., 2018; Valcek et al., 2019), revealed high genetic similarity. Thus, there is reason to believe that highly similar IncI1-Iγ/ST3 plasmids are widespread and successful in the European broiler production. This has also been reported for other plasmids encoding ESC-resistance in broiler production (Hansen et al., 2016; Mo et al., 2016), and is consistent with a common source (Dierikx C.M. et al., 2013; Agersø et al., 2014; Nilsson et al., 2014; Zurfluh et al., 2014). Reconstruction of IncI1-Iγ/ST3 plasmids also revealed some genetic diversity. All shared a common backbone, but some differences were observed, especially in the accessory module. Furthermore, some amino acid differences were observed in regions encoding replication, partitioning, entry exclusion, TrbABC and NikB. This indicates a certain flux of genes at the plasmid level, and further studies are required to determine whether this has an effect on plasmid stability and/or conjugation frequency.

We detected only three IncI1-Iγ plasmids belonging to ST7 in our isolates, all of which were non-transferable. A disruption of the *traC* gene, known to be essential for conjugation (Kim et al., 1993), was the most likely cause of the non-conjugative phenotype. This could also explain why we only detected them in a limited number of isolates and a single *E. coli* ST. Thus, it seems that ST7 plasmids are of limited epidemiological importance in Norwegian broiler production, although they have been reported to have high epidemiological relevance internationally (Carattoli et al., 2018). Furthermore, all ST3 plasmids characterized in our study encoded a colicin. Colicin production has been reported to represent a selective advantage, as it can have lethal effect on related bacteria in the gut without colicin production (Cascales et al., 2007; Majeed et al., 2011). It is also plausible that colicin production together with the presence of plasmid-encoded addiction systems, will facilitate persistence of IncI1-Iγ/*bla*_CTX–M–__1_ plasmids in an *E. coli* population despite the lack of selection pressure from antimicrobial use, which is the case in Norwegian broiler production (NORM/NORM-VET, 2019).

One of the reconstructed plasmids (p17437) turned out to be a co-integrated plasmid harboring both an IncI1-Iγ and an IncFIB replicon. The IncFIB specific part was inserted into the accessory module. Co-integrated IncI1/IncFIB plasmids have been described in a few previous studies in *Salmonella* and enterotoxigenic *E. coli* (ETEC) (Froehlich et al., 2005; Johnson and Nolan, 2009; Crossman et al., 2010; Moreno Switt et al., 2012). It has also been suggested that such human ETEC plasmids might have an animal origin (Johnson et al., 2011). The co-integrated plasmids found in *Salmonella* (Moreno Switt et al., 2012) had the IncF region inserted in a similar manner as described in this study, adjacent to the IncI replicon *rep*Z. The IncFIB fraction of our co-integrated plasmid harbored several virulence genes. This could represent a selective advantage, as the plasmid will confer both virulence and antimicrobial resistance, but this hypothesis needs to be investigated further. Interestingly, we only detected the co-integrated IncI1-Iγ/IncFIB plasmid in a single ST, namely ST57. This was also the most commonly occurring ST in our material, detected from a total of six different farms. All but one of the ST57 transconjugants were positive for both the IncI1-Iγ and IncFIB replicons. This was confirmed by plasmid profiling, as all but one of the transconjugants from ST57 isolates harbored a large plasmid with a size corresponding to the co-integrative plasmid. One ST3 plasmid (p19138) differed from the others in the regions encoding *traABC* and *nikB*. Interestingly, this plasmid was only detected in *E. coli* ST297. This was the second most common ST in our material, isolated from six different farms. These two examples could indicate successful plasmid-host combinations, and should be further investigated. The systematic and comprehensive sampling behind our dataset can provide support for some trends at the plasmid level. We found that IncI1-Iγ/ST3 plasmids were more common than IncI1-Iγ/ST7 plasmids. A contributing factor could be that the ST7 plasmids were non-conjugative, thus having a lower potential for dissemination in the bacterial population. Furthermore, the clonal dissemination of both ST57 and ST297 can partly explain the dominance of ST3 plasmids in our material. In addition, our dataset enabled comparison of IncI1-Iγ/ST3 plasmids occurring in different *E. coli* STs. The comparison showed highly conserved backbones, but also variations. This demonstrated dissemination of similar, but distinguishable ST3 plasmids. To a wide extent, the observed variations were associated with the accessory region, which is in concordance with previous findings (Johnson et al., 2011). The observed differences were mainly due to different compositions of AMR genes. In addition, smaller variations were observed on other locations of the plasmid. This further supports the observation by Smith et al. (2015) that accessory genes can integrate on several locations in IncI1-Iγ plasmids.

## Conclusion

In conclusion, our results point toward a scenario where both clonal dissemination and horizontal transfer of plasmids have contributed to the dissemination of *bla*_CTX–M–__1_ in Norway. Our studies revealed circulation of similar plasmids, but some genetic diversity was present among the IncI1-Iγ/*bla*_CTX–M–__1_ plasmids recovered during the sampling period. The plasmids represented variants grouping into two main lineages, namely CC-3 and CC-7, described from broiler production in several countries. The genetic diversity indicates multiple introductions to the Norwegian broiler production. The close genetic relationship between plasmids originating from broiler production in Norway and other European countries, provides further evidence for the theory of a common source of the ESC-resistant isolates.

## Data Availability Statement

Plasmid sequences are uploaded to NCBI; MN419430, MN419431, MN419432, MN419433, MN419434, MN419435, MN419436, MN419437, and MN419438. Raw reads from all isolates will be submitted to ENA and accession numbers reported accordingly.

## Author Contributions

SM, AT, and MS contributed to the conception and design of the study, while AU was responsible for the conception and design of the study the isolates originated from. SM, AT, KO, HI, and TL performed the laboratory work. SM, AT, KO, JS, CS, and MS performed the *in silico* analyses. SM wrote the first draft of the manuscript. AT and MS wrote sections of the manuscript. All authors contributed to manuscript revision, read and approved the submitted version.

## Conflict of Interest

The authors declare that the research was conducted in the absence of any commercial or financial relationships that could be construed as a potential conflict of interest.

## Footnotes

1 https://github.com/NorwegianVeterinaryInstitute/Bifrost

2 https://www.bioinformatics.babraham.ac.uk/projects/fastqc/

3 https://sourceforge.net/projects/bbmap/

4 https://enterobase.warwick.ac.uk/

5 https://github.com/rrwick/Filtlong.git

6 http://www.ncbi.nlm.nih.gov
