# Corrigendum: *bla*_**CTX–M–1**_/IncI1-Iγ Plasmids Circulating in *Escherichia coli* From Norwegian Broiler Production Are Related, but Distinguishable

**DOI:** 10.3389/fmicb.2021.614185

**Published:** 2021-03-24

**Authors:** Solveig Sølverød Mo, Amar Anandrao Telke, Kingsley Oteng Osei, Camilla Sekse, Jannice Schau Slettemeås, Anne Margrete Urdahl, Hanna Karin Ilag, Thongpan Leangapichart, Marianne Sunde

**Affiliations:** ^1^Section for Food Safety and Animal Health Research, Department of Animal Helath, Welfare and Food Safety, Norwegian Veterinary Institute, Oslo, Norway; ^2^Faculty of Chemistry, Biotechnology and Food Science, Norwegian University of Life Sciences, Ås, Norway; ^3^Section for Microbiology, Department of Analysis and Diagnostics, Norwegian Veterinary Institute, Oslo, Norway

**Keywords:** plasmid, IncI1, cephalosporin resistance, *Escherichia coli*, broiler, *bla*_CTX–M–1_

In the original article, the reference for The Bifrost pipeline was incorrectly written as ‘commitid 0f2ef57’. It should instead be written as ‘Lagesen, 2020’ in the text, with the reference below added to the reference list:

Lagesen, K. (2020). *NorwegianVeterinaryInstitute/Bifrost: v1.1 (Version v1.1)*. Zenodo. doi: 10.5281/zenodo.4020511

The authors apologize for this error and state that this does not change the scientific conclusions of the article in any way. The original article has been updated.
