# Corrigendum: *bla*_CTX–M–1_/IncI1-Iγ Plasmids Circulating in *Escherichia coli* From Norwegian Broiler Production Are Related, but Distinguishable

**DOI:** 10.3389/fmicb.2021.730152

**Published:** 2021-07-16

**Authors:** Solveig Sølverød Mo, Amar Anandrao Telke, Kingsley Oteng Osei, Camilla Sekse, Jannice Schau Slettemeås, Anne Margrete Urdahl, Hanna Karin Ilag, Thongpan Leangapichart, Marianne Sunde

**Affiliations:** ^1^Section for Food Safety and Animal Health Research, Department of Animal Health, Welfare and Food Safety, Norwegian Veterinary Institute, Oslo, Norway; ^2^Faculty of Chemistry, Biotechnology and Food Science, Norwegian University of Life Sciences, Ås, Norway; ^3^Section for Microbiology, Department of Analysis and Diagnostics, Norwegian Veterinary Institute, Oslo, Norway

**Keywords:** plasmid, IncI1, cephalosporin resistance, *Escherichia coli*, broiler, *bla*_CTX–M–1_

In the published article, there was an error in affiliation **1**. Instead of “Section for Food Safety and Animal Health Research, Department of Animal Helath, Welfare and Food Safety, Norwegian Veterinary Institute, Oslo, Norway,” it should be “Section for Food Safety and Animal Health Research, Department of Animal Health, Welfare and Food Safety, Norwegian Veterinary Institute, Oslo, Norway.”

In the original article, there was an error. The accession number for the raw Illumina reads of the 31 *E. coli* isolates was missing.

A correction has been made to Materials and Methods, Sequence Analysis and Characterization, paragraph number 4.

Complete plasmid sequences are available in GenBank; MN419430 (p17437), MN419431 (p19138), MN419432 (p21254), MN419433 (p20426), MN419434 (21249), MN419435 (p14263), MN419436 (p22440), MN419437 (p22638), and MN419438 (p24003). Raw reads for the 31 *E. coli* isolates are available from ENA (bioproject number PRJEB45077).

The authors apologize for this error and state that this does not change the scientific conclusions of the article in any way. The original article has been updated.

