# Supplementary material for: blaCTX–M–1/IncI1-Iγ Plasmids Circulating in Escherichia coli From Norwegian Broiler Production Are Related, but Distinguishable
Source: Front Microbiol. 2020 Mar 5;11:333. doi: 10.3389/fmicb.2020.00333 (PMC7066084; doi:10.3389/fmicb.2020.00333)
Supplement: Supplementary file 1 [file Data_Sheet_1.PDF]

Supplementary material

1 Supplementary Figures and Tables

1.1 Supplementary Tables

Supplementary Table 1. Overview of virulence genes present in 31 *Escherichia coli* isolates with *bla*$_{CTX-M-1}$ isolated from Norwegian broiler production in 2016. Virulence genes were predicted using ARIBA (antimicrobial resistance identification by assembly) and the VirulenceFinder database.

ID	*astA*	*cif*	*cma*	*eae*	*espA*	*espB*	*espF*	*gad*	*iha*	*iroN*	*iss*	*lpfA*	*mchB*	*mchC*	*mchF*	*nleB*	*tir*	*tsh*	Pathogroup (diarrhoeagenic)
2016-40-14263	0	1	0	1	1	1	0	0	0	1	1	0	0	0	1	1	1	1	EPEC
2016-40-14272	0	0	1	0	0	0	0	0	0	1	1	0	0	0	0	0	0	0	
2016-40-14497	0	0	1	0	0	0	0	0	0	1	1	0	0	0	0	0	0	0	
2016-40-16262	0	0	1	0	0	0	0	0	0	1	1	0	0	0	0	0	0	0	
2016-40-16344	0	0	1	0	0	0	0	1	0	1	1	0	0	0	0	0	0	0	
2016-40-16990	0	0	1	0	0	0	0	0	0	1	1	0	0	0	0	0	0	0	
2016-40-17074	0	0	1	0	0	0	0	0	0	1	1	0	0	0	0	0	0	0	
2016-40-17091	0	0	1	0	0	0	0	0	0	1	1	0	0	0	0	0	0	0	
2016-40-17200	0	0	1	0	0	0	0	1	0	1	1	0	0	0	0	0	0	0	
2016-40-17381	0	0	0	0	0	0	0	1	1	1	1	1	1	1	1	0	0	1	
2016-40-17437	0	0	1	0	0	0	0	0	0	1	1	0	0	0	0	0	0	0	
2016-40-19016	0	0	0	0	0	0	0	1	1	1	1	1	1	1	1	0	0	1	
2016-40-19138	0	0	0	0	0	0	0	0	1	1	1	1	1	1	0	0	0	1	
2016-40-19148	0	0	0	0	0	0	0	0	1	1	1	1	1	1	1	0	0	1	
2016-40-19583	0	0	1	0	0	0	0	0	0	1	1	0	0	0	0	0	0	0	
2016-40-19738	0	0	1	0	0	0	0	0	0	1	1	0	0	0	0	0	0	0	
2016-40-19970	0	0	0	0	0	0	0	1	1	1	1	1	1	1	1	0	0	1	

2016-40-20426	0	0	0	0	0	0	0	0	0	0	0	1	0	0	0	0	0	0	
2016-40-20481	0	0	1	0	0	0	0	0	0	1	1	0	0	0	0	0	0	0	
2016-40-20703	0	0	0	1	1	1	0	0	0	1	1	0	0	0	0	0	1	1	EPEC
2016-40-21210	0	0	0	0	0	0	0	0	1	1	1	1	1	1	0	0	0	1	
2016-40-21249	0	0	0	0	0	0	0	1	0	1	0	1	0	0	1	0	0	0	
2016-40-21254	1	0	0	0	0	0	0	0	0	0	1	0	0	0	0	0	0	0	
2016-40-21270	0	0	0	0	0	0	0	1	1	1	1	1	1	1	1	0	0	1	
2016-40-22440	0	0	1	0	0	0	0	0	0	1	0	0	0	0	0	0	0	0	
2016-40-22638	0	0	1	0	0	0	0	1	1	1	1	0	1	1	1	0	0	0	
2016-40-23572	0	0	1	0	0	0	0	1	0	1	0	0	0	0	0	0	0	0	
2016-40-23574	0	0	1	0	0	0	0	1	1	1	1	0	1	1	1	0	0	0	
2016-40-23575	0	1	0	1	1	1	1	1	0	1	1	0	0	0	0	1	1	1	EPEC
2016-40-24003	0	0	1	0	0	0	0	0	0	1	1	0	0	0	0	0	0	0	
2016-40-24053	0	0	1	0	0	0	0	0	0	1	0	0	0	0	0	0	0	0	
SUM	1	2	18	3	3	3	1	11	9	29	26	9	9	9	9	2	3	10	

1.2 Supplementary Figures

```
p20426  -ATTCTGTGAGGCCCCCACTATCTTTCGTCGTTCCGCCAAGTTCGTAAGAAAGATAGTGGGGGTTTTCCTTTT 72
   R64  -ATTCTGTGAGGCCCCCACTATCTTTCGTCGTTCCGCCAAGTTCGTAAGAAAGATAGTGGGGGTTTTCCTTTT 72
p19138  -ATTCTGTGAGGCCCCCACTATCTTTCGTCGTTCCGCCAAGTTCGTAAGAAAGATAGTGGGGGTTTTCCTTTT 72
p21249  -ATTCTGTGAGGCCCCCACTATCTTTCGTCGTTCCGCCAAGTTCGTAAGAAAGATAGTGGGGGTTTTCCTTTT 72
p22440  -ATTCTGTGAGGCCCCCACTATCTTTCGTCGTTCCGCCAAGTTCGTAAGAAAGATAGTGGGGGTTTTCCTTTT 72
p21254  -ATTCTGTGAGGCCCCCACTATCTTTCGTCGTTCCGCCAAGTTCGTAAGAAAGATAGTGGGGGTTTTCCTTTT 72
p22638  -ATTCTGTGAGGCCCCCACTATCTTTCGTCGTTCCGCCAAGTTCGTAAGAAAGATAGTGGGGGTTTTCCTTTT 72
p14263  -ATTCTGTGAGGCCCCCACTATCTTTCGTCGTTCCGCCAAGTTCGTAAGAAAGATAGTGGGGGTTTTCCTTTT 72
p24003  -ATTCTGTGAGGCCCCCACTATCTTTCGTCGTTCCGCCAAGTTCGTAAGAAAGATAGTGGGGGTTTTCCTTTT 72
p17437  -ATTCTGTGAGGCCCCCACTATCTTTCGTCGTTCCGCCAAGTTCGTAAGAAAGATAGTGGGGGTT---CTTTT 69
 R621a  TATTCTGTGAGGCCCCCATAATCTTTCTCCGTCCCGCCAAGTTCGTC-GAAAGATTGTGGGGGTTTTCCTTTT 72
```

Supplementary Figure 1. Alignment of the nucleotide sequences of *inc* of p14263, p20426, p21249, p21254, p19138, p22440, p24003, p22638 and p17437 including comparison to the IncI1 reference plasmid R64 (accession number AP005147) and Inc1γ reference plasmid R621a (accession number AP011954.1). Gaps are marked by dashes and nucleotide differences shown in blue.

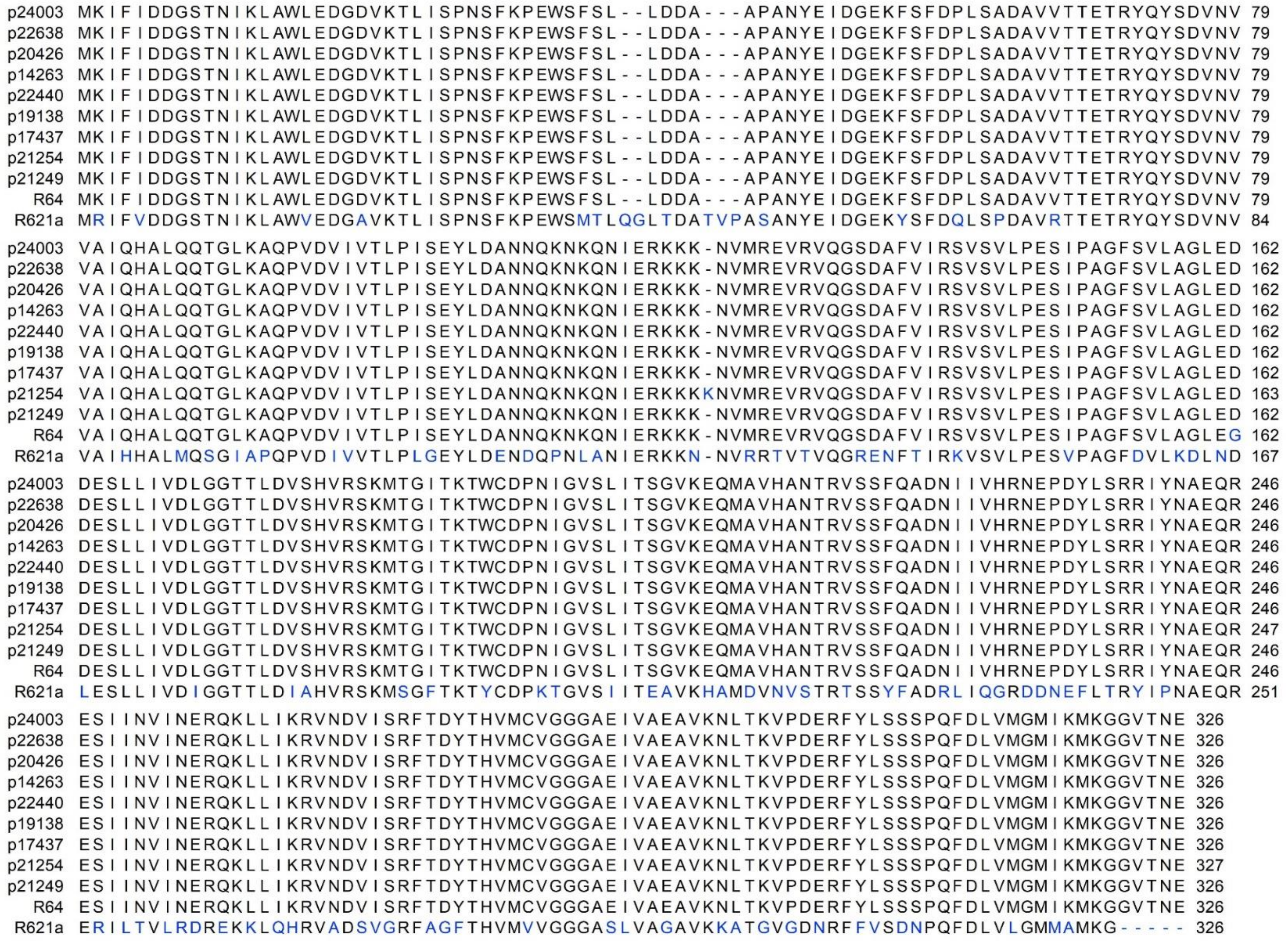

Supplementary Figure 2. Alignment of the amino acid sequences of ParA of p14263, p20426, p21249, p21254, p19138, p22440, p24003, p22638 and p17437 including comparison to the IncI1 reference plasmid R64 (accession number AP005147) and Inc1γ reference plasmid R621a (accession number AP011954.1). Gaps are marked by dashes and amino acid differences shown in blue.

```
p24003  MSDENKSRRCSFELFPDERTGDKIADELIANEKLKERGRFMRAMLVTGAAFAAIDKRLPLLISELLTENTTLDDINKVISSVIP 84
p22638  MSDENKSRRCSFELFPDERTGDKIADELIANEKLKERGRFMRAMLVTGAAFAAIDKRLPLLISELLTENTTLDDINKVISSVIP 84
p20426  MSDENKSRRCSFELFPDERTGDKIADELIANEKLKERGRFMRAMLVTGAAFAAIDKRLPLLISELLTENTTLDDINKVISSVIP 84
p14263  MSDENKSRRCSFELFPDERTGDKIADELIANEKLKERGRFMRAMLVTGAAFAAIDKRLPLLISELLTENTTLDDINKVISSVIP 84
p22440  MSDENKSRRCSFELFPDERTGDKIADELIANEKLKERGRFMRAMLVTGAAFAAIDKRLPLLISELLTENTTLDDINKVISSVIP 84
p19138  MSDENKSRRCSFELFPDERTGDKIADELIANEKLKERGRFMRAMLVTGAAFAAIDKRLPLLISELLTENTTLDDINKVISSVIP 84
p21249  MSDENKSRRCSFELFPDERTGDKIADELIANEKLKERGRFMRAMLVTGAAFAAIDKRLPLLISELLTENTTLDDINKVISSVIP 84
p17437  MSDENKSRRCSFELFPDERTGDKIADELIANEKLKERGRFMRAMLVTGAAFAAIDKRLPLLISELLTENTTLDDINKVISSVIP 84
p21254  MSDENKSRRCSFELFPDERTGDKIADELIANEKLKERGRFMRAMLVTGAAFAAIDKRLPLLISELLTENTTLDDINKVISSVIP 84
R64     MSDENKSRRCSFELFPDERTGDKIADELIANEKLKERGRFMRAMLVTGAAFAAIDKRLPLLISELLTENTTLDDINKVISSVIP 84
621a    MAD---NRKCSFYIYPERNAADRVADRFLEKLPQKERGRAMRAMMLCGAALMKQDERLPFLIAEFLTDSTSMQDIQRIISSTLP 81

p24003  GAFSVEK-KLLELLEKQSGLHT-----SVDCSTPLTEQSLSRNDGEDQTRRNAENMFGDD- 138
p22638  GAFSVEK-KLLELLEKQSGLHT-----SVDCSTPLTEQSLSRNDGEDQTRRNAENMFGDD- 138
p20426  GAFSVEK-KLLELLEKQSGLHT-----SVDCSTPLTEQSLSRNDGEDQTRRNAENMFGDD- 138
p14263  GAFSVEK-KLLELLEKQSGLHT-----SVDCSTPLTEQSLSRNDGEDQTRRNAENMFGDD- 138
p22440  GAFSVEK-KLLELLEKQSGLHT-----SVDCSTPLTEQSLSRNDGEDQTRRNAENMFGDD- 138
p19138  GAFSVEK-KLLELLEKQSGLHT-----SVDCSTPLTEQSLSRNDGEDQTRRNAENMFGDD- 138
p21249  GAFSVEK-KLLELLEKQSGLHT-----SVDCSTPLTEQSLSRNDGEDQTRRNAENMFGDD- 138
p17437  GAFSVEK-KLLELLEKQSGLHT-----SVDCSTPLTEQSLSRNDGEDQTRRNAENMFGDD- 138
p21254  GAFSVEK-KLLELLEKQSGLHT-----SVDCSTPLTEQSLSRNDGEDQTRRNAENMFGDD- 138
R64     GAFSVEK-KLLELLEKQSGLHT-----SVDCSTPLTEQSLSRNDGEDQTRRNAENMFGDD- 138
621a    QQENGEVVRLLEAFLQSAGNNAKAILPAVDSATQEISAPVDQNLLE--TRNNIKNLFPDDE 140
```

Supplementary Figure 3. Alignment of the amino acid sequences of ParB of p14263, p20426, p21249, p21254, p19138, p22440, p24003, p22638 and p17437 including comparison to the IncI1 reference plasmid R64 (accession number AP005147) and Inc1γ reference plasmid R621a (accession number AP005147). Gaps are marked by dashes and amino acid differences shown in blue.

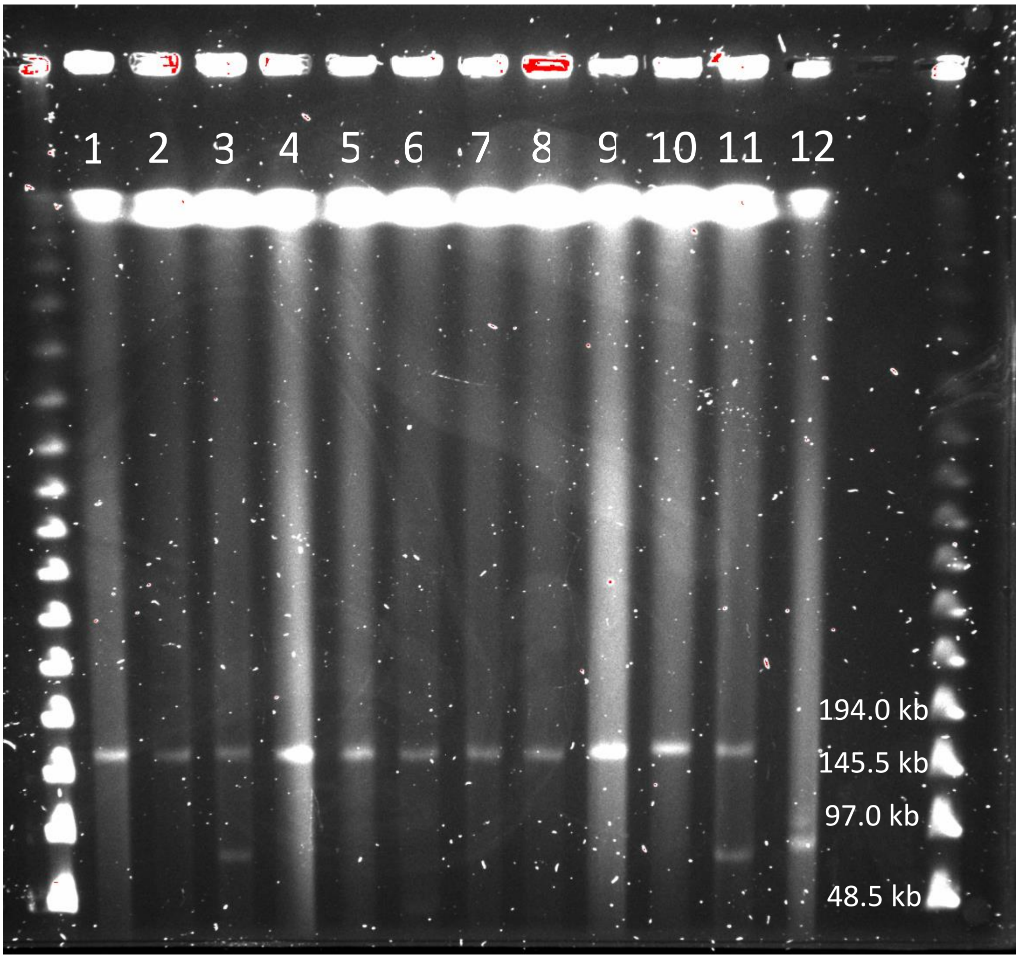

Supplementary Figure 4. Gel displaying plasmid content in 12 transconjugants after digestion with S1 nuclease. Isolates 1-11 were positive for both IncI1-1γ and IncFIB replicons, while isolate 12 only had the IncI1-Iγ replicon. The isolates are transconjugants of the following isolates; 1:2016-40-14272, 2:2016-40-14497, 3:2016-40-16262, 4:2016-40-16344, 5:2016-40-16990, 6:2016-40-17074, 7:2016-40-17091, 8:2016-40-17200, 9:2016-40-17437, 10:2016-40-19583, 11:2016-40-19738, 12:2016-40-20481.

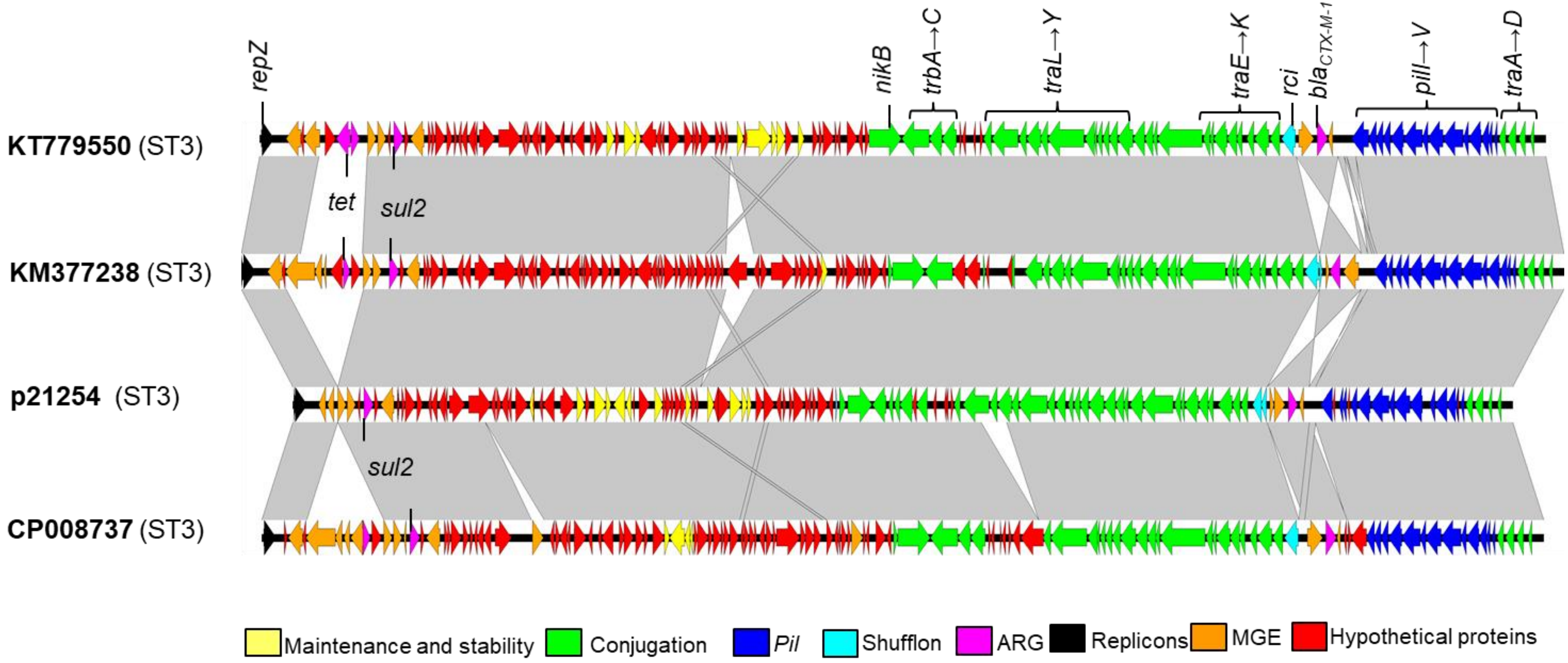

Supplementary Figure 5. Comparison of three previously published IncI1-Iγ/ST3 plasmids from broiler production in France (accession number KT779550, Baron et al. 2016), Switzerland (accession number KM377238, Zurfluh et al. 2014) and the Netherlands (accession number CP008737, Brouwer et al. 2014) and one from Norway (p21254, this study).

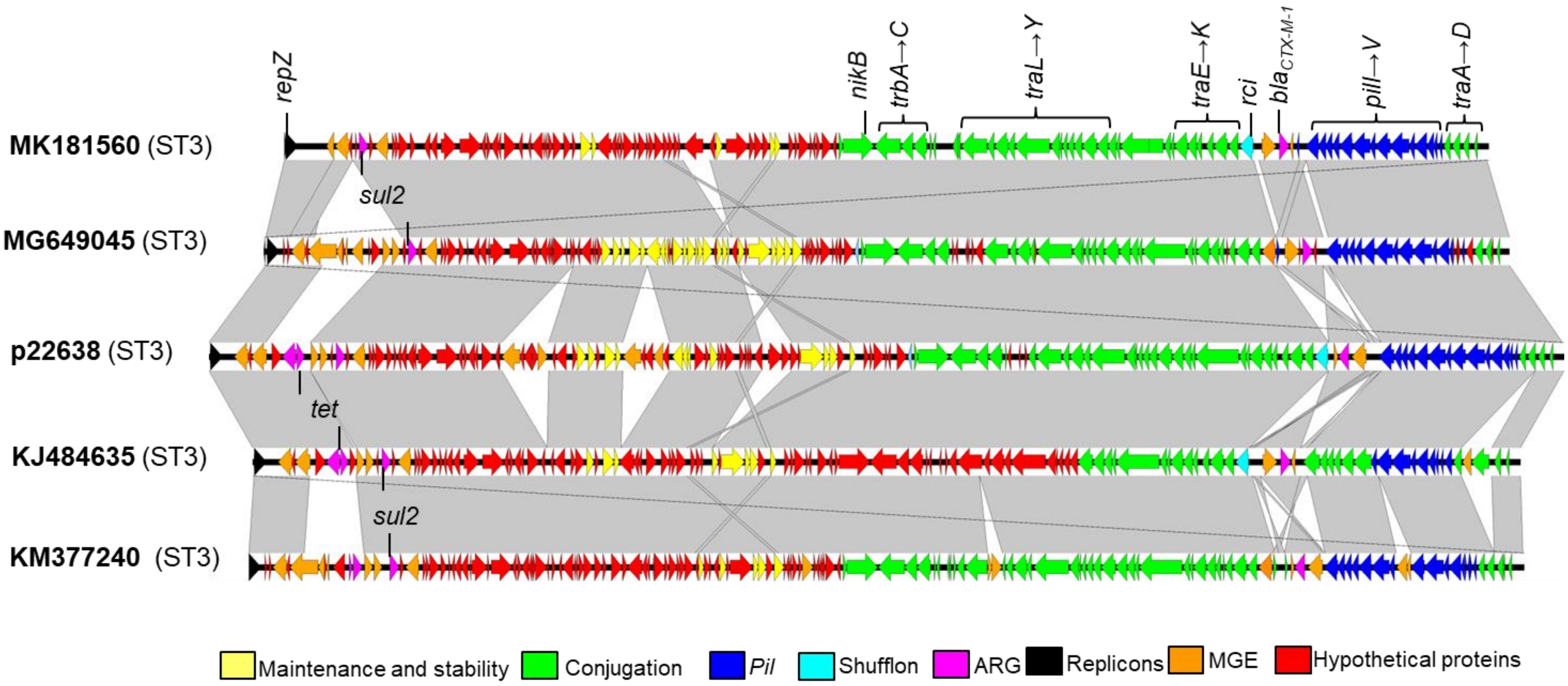

Supplementary Figure 6. Comparison of four previously published IncI1-Iγ/ST3 plasmids from broiler production in Denmark (accession number MK181560, Valcek et al. 2019), France (accession number MG649045, Touzain et al. 2018), Switzerland (accession number KJ484635, Wang et al. 2014 and KM377240, Zurfluh et al. 2014) and one from Norway (p22638, this study).

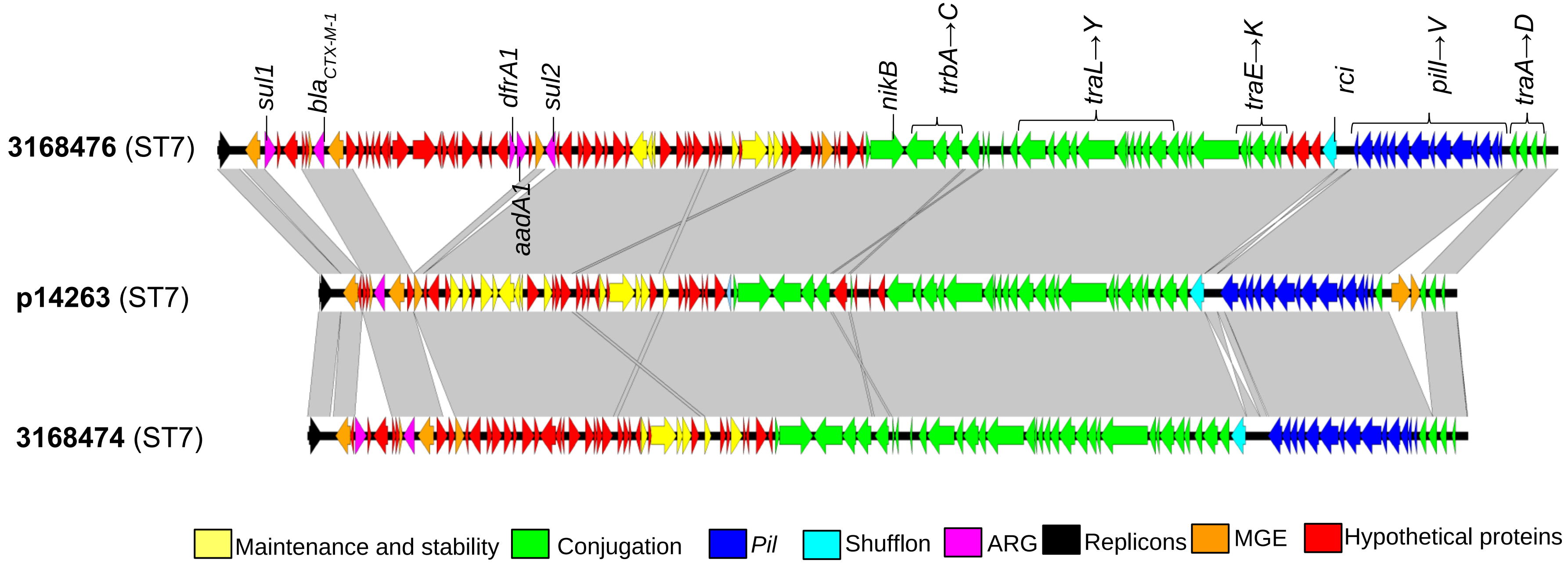

Supplementary Figure 7. Comparison of two previously published IncI1-Iγ/ST7 plasmids from poultry in the Netherlands (accession numbers 3168476 and 3168474, Smith et al. 2015) and one from Norway (p14263, this study).

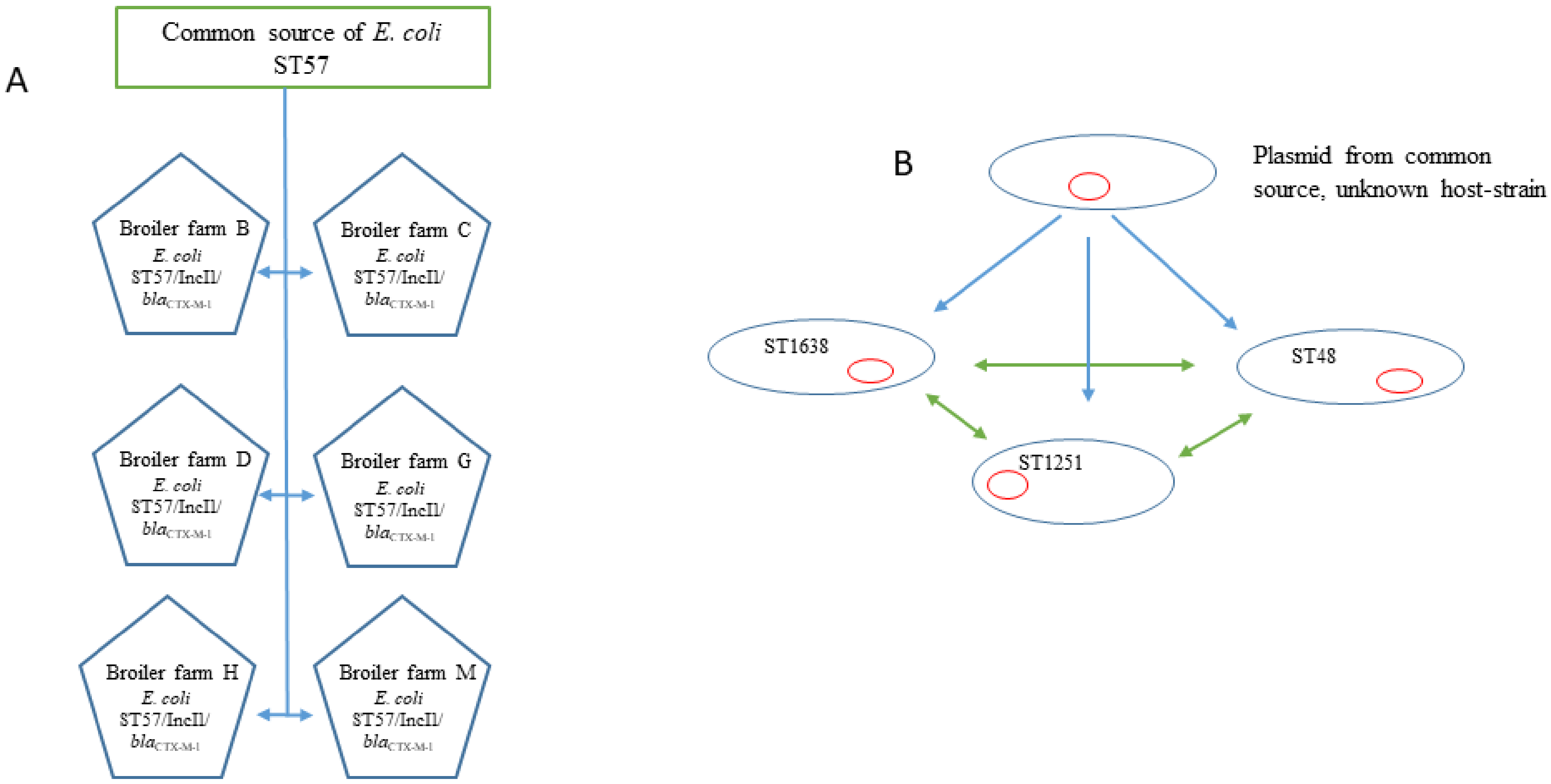

Supplementary Figure 8. Visualization of A) clonal dissemination of *E. coli* ST57 carrying *bla*$_{CTX\text{-}M\text{-}1}$ on an IncI1-Iγ plasmid in the broiler production and B) horizontal transfer of an IncI1-Iγ plasmid with *bla*$_{CTX\text{-}M\text{-}1}$ from one *E. coli* sequence type to another. Highly similar IncI1 plasmids were detected in different *E. coli* STs (see also Figure 2), indicating horizontal dissemination of the IncI1-Iγ plasmid.
